# Resource for FRET-Based Biosensor Optimization

**DOI:** 10.3389/fcell.2022.885394

**Published:** 2022-06-20

**Authors:** Heonsu Kim, Gyuho Choi, Myung Eun Suk, Tae-Jin Kim

**Affiliations:** ^1^ Institute of Systems Biology, Pusan National University, Busan, South Korea; ^2^ Department of Integrated Biological Science, Pusan National University, Busan, South Korea; ^3^ Department of Mechanical Engineering, IT Convergence College of Materials and Components Engineering, Dong-Eui University, Busan, South Korea; ^4^ Department of Biological Sciences, Pusan National University, Busan, South Korea

**Keywords:** genetically encoded biosensor, optimization, FRET, sensor domain, ligand domain, linker, localization signal

## Abstract

After the development of Cameleon, the first fluorescence resonance energy transfer (FRET)-based calcium indicator, a variety of FRET-based genetically encoded biosensors (GEBs) have visualized numerous target players to monitor their cell physiological dynamics spatiotemporally. Many attempts have been made to optimize GEBs, which require labor-intensive effort, novel approaches, and precedents to develop more sensitive and versatile biosensors. However, researchers face considerable trial and error in upgrading biosensors because examples and methods of improving FRET-based GEBs are not well documented. In this review, we organize various optimization strategies after assembling the existing cases in which the non-fluorescent components of biosensors are upgraded. In addition, promising areas to which optimized biosensors can be applied are briefly discussed. Therefore, this review could serve as a resource for researchers attempting FRET-based GEB optimization.

## 1 Introduction

After successful cloning of green fluorescent protein (GFP) originating from *Aequorea victoria* and having it exogenously expressed in cells (Morin and Hastings, 1971; Prasher et al., 1992), researchers have developed genetically encoded biosensors (GEBs) using fluorescent proteins (FPs). These GEBs allow visualization of various cellular biochemical parameters, such as ion concentration, cellular properties, and enzymatic activity (Miyawaki et al., 1997; Zhang et al., 2001; Wang et al., 2005). GEB is a chimeric protein expressed by transduction of an expression vector into cells and consists of organic fluorescent materials such as FPs or bioluminescent proteins and various components that induce the function of the biosensor (Sanford and Palmer, 2017; Greenwald et al., 2018; Terai et al., 2019). The advantage of GEB is that it directly interacts with endogenous players in cells and spatiotemporally visualizes intra- and extra-cellular properties. In addition, because it contains fluorescent proteins, chemical dyes inducing cytotoxicity are not required, and GEBs are observed and analyzed using microscopic imaging modalities or microplate readers. To date, researchers have developed biosensors in various categories based on circularly permuted FP, dimerization-dependent FP, reconstitution of split FP, bioluminescence resonance energy transfer (BRET), and fluorescence resonance energy transfer (FRET) (Sanford and Palmer, 2017; Kim et al., 2021). In this review, we mainly deal with FRET-based GEBs in particular.

**FIGURE 1:**
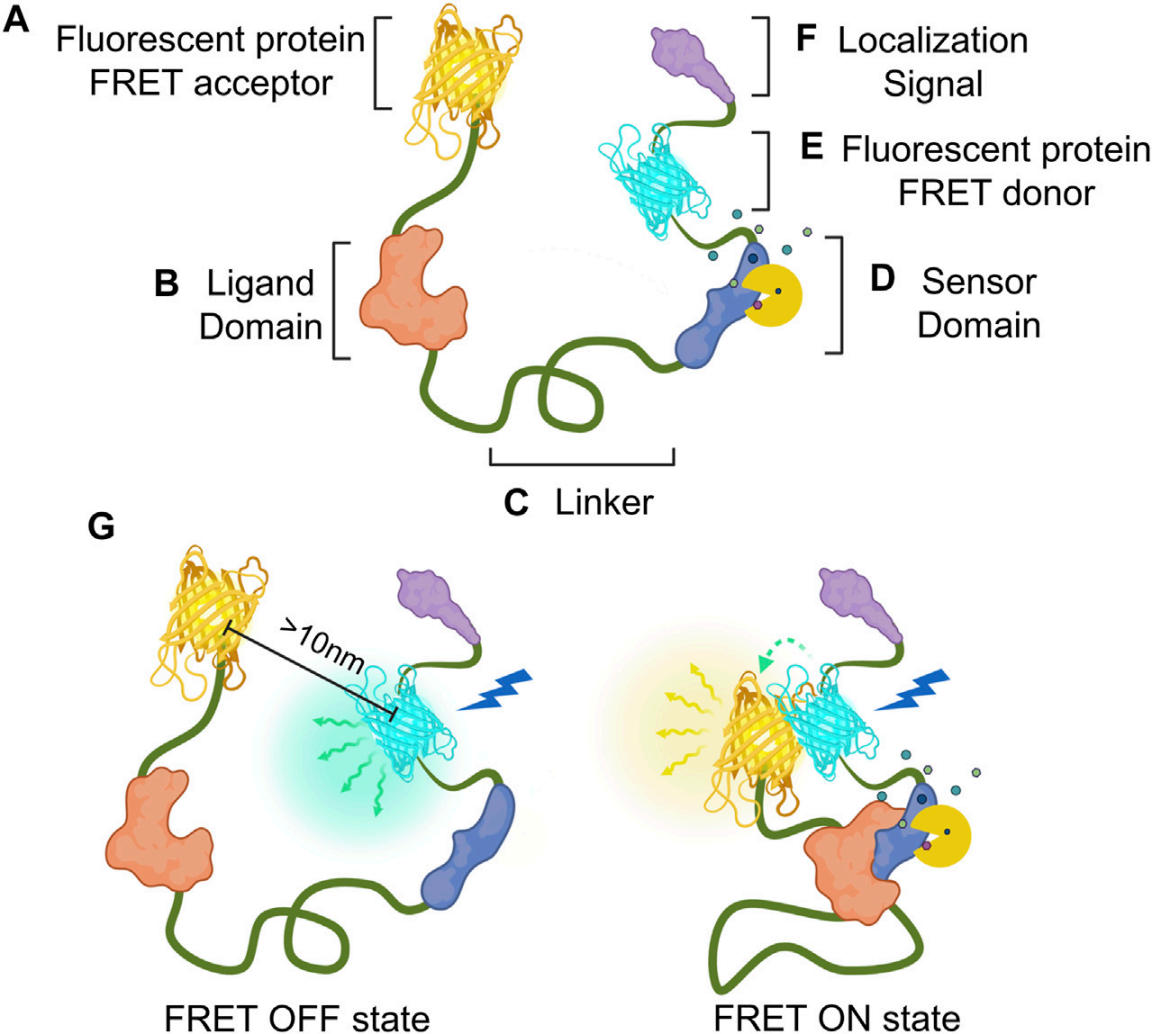
Composition and mechanism of FRET-based GEB. Genetically encoded biosensor (GEB) consists of the following components. **(A,E)** Fluorescent proteins for acting as acceptor and donor, respectively; **(B)** ligand domain for recognizing the conformational change of the sensor domain; **(C)** linker for connecting the components of the GEB, which can affect the structure and performance of GEB; **(D)** sensor domain for detecting researcher’s target of interest such as enzyme activity (yellow Pacman shape) or target molecule (small polygons); and **(F)** localization signal for placing GEBs in specific microdomains in a cell. **(G)** Mechanism of FRET-based GEB on FRET OFF state and FRET ON state. In the FRET OFF state, because ligand and sensor domain are not interacting with each other, donor and acceptor fluorescent protein generally keep a distance longer than 10 nm. Therefore, donor emission (green winding arrow and blue-green emanating glow) can be mainly detected by donor excitation (blue thunder shape). But In the FRET ON state, since GEB is activated by a specific target signal, The interaction between ligand and sensor domain makes two fluorescent proteins close. Due to this FRET phenomenon (green dashed-fading arrow), acceptor emission (yellow winding arrow and yellow emanating glow) can be mainly detected by donor excitation.

FRET is a physical phenomenon of non-radiative energy transfer between two close chromophores with spectral overlap; an emission spectrum of donor fluorophore overlaps an excitation spectrum of acceptor fluorophore (Förster, 1948; Jares-Erijman and Jovin, 2003). When a donor fluorophore absorbs excitation light, the donor transfers its energy to a neighboring acceptor fluorophore no farther than 10 nm, which results in the emission of the acceptor fluorophore and FRET ON state (Figure 1G). As FRET is a precise phenomenon that occurs between close molecules, it has now been widely used in biology as a tool to detect the interaction and proximity of two proteins. Since the development of the first genetically encoded calcium indicator (GECI) based on the FRET phenomenon (Miyawaki et al., 1997), many researchers have developed FRET-based GEB to monitor various target molecules and optimized biosensors to measure the activities of the targets of interest more accurately and sensitively.

Researchers have mainly optimized their biosensors using the following strategies. The first tactic is to adapt the improved FPs to their GEBs. The early FPs constituting the FRET pair, mainly cyan fluorescent protein (CFP) and yellow fluorescent protein (YFP), have many disadvantages. The acceptor YFP has poor resistance to low pH and photostability (Griesbeck et al., 2001; Nagai et al., 2002). In the case of CFP, the quantum yield, which is the degree to efficiently emit photons in response to excitation, had to be improved for better FRET efficiency (Rizzo et al., 2004; Goedhart et al., 2010; Goedhart et al., 2012). Many researchers have reported refined FPs, and FRET-exclusive FPs that allow the FRET pair to form dimers have been developed to stabilize the basal FRET signal (Nguyen and Daugherty, 2005). Applying these upgraded FPs to GEBs improved the FRET efficiency radiated by the biosensors (Allen and Zhang, 2006; Komatsu et al., 2011). Because there are several excellent review papers on FP development (Tsien, 1998; Zimmer, 2002; Chudakov et al., 2010), we do not focus on this topic in this article. The second upgrade strategy involves rearranging the order of the components constituting the GEBs. Two FPs of Mermaid 1, a voltage sensor, were located at the C-terminal of the amino acid sequence in a row (Tsutsui et al., 2008). However, Mermaid 2, developed by Tsutsui et al., showed an enhanced FRET ratio change by placing each FP in the N- and C-termini of the sensor domain, respectively (Tsutsui et al., 2013). The last strategy is to structurally analyze that, except for fluorescent proteins, the remaining components constituting the biosensor interact with the target molecules or other components; thereafter, each component is manipulated based on previous studies (Palmer et al., 2006). This process requires knowledge of other researchers’ ideas, peptide domains that have been previously used and improved, and numerous examples of biosensor optimizations. However, discussions on strategies and cases in which researchers have optimized the components of GEBs are still lacking.

Here, we introduce several optimization strategies based on how the non-fluorescent components of FRET-based GEBs were refined by many researchers. At the end of this review, there is a brief introduction to fields where biosensors were newly applied as GEBs have been improved. Before starting this review, we define the components that compose a biosensor and have been optimized as follows (Figures 1A–F). The sensor domain plays a role in translating the property of the observer’s interest into the appropriate conformational change of the biosensor (Figure 1D). The ligand domain recognizes conformational changes in the sensor domain (Figure 1B). The linker is the connection between components that affect the structure and performance of the GEBs (Figure 1C). Localization signals are peptides that can place the GEBs in specific subcellular locations (Figure 1F).

## 2 Sensor Domain

**FIGURE 2:**
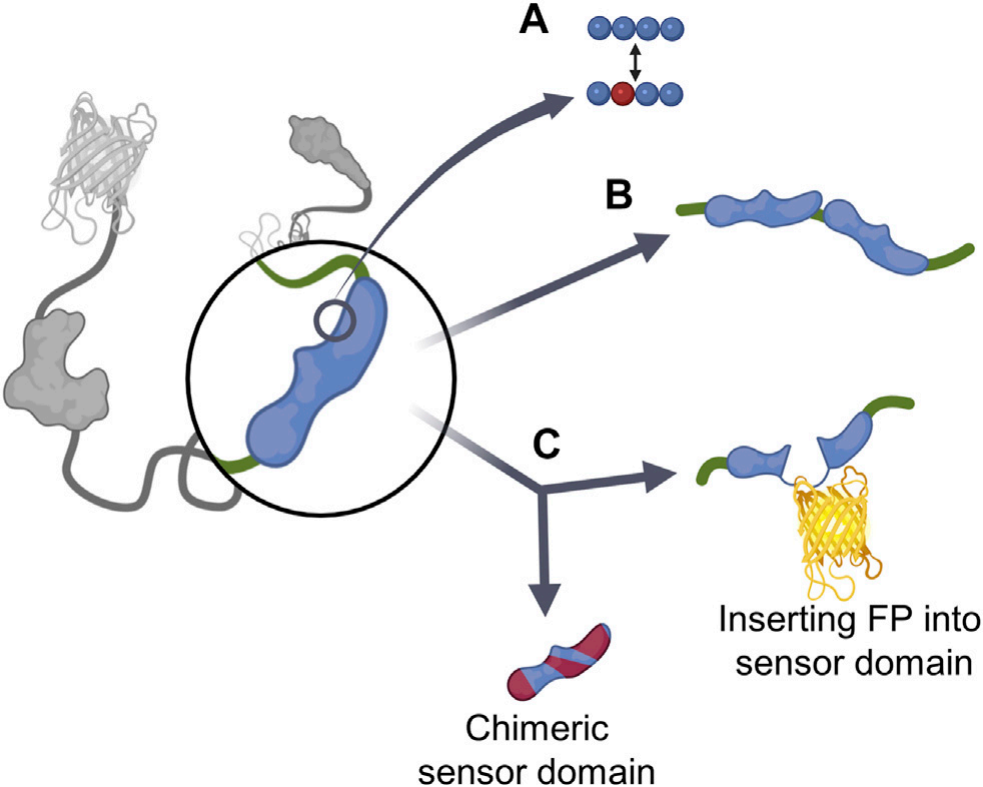
Existing cases of sensor domain optimization strategies **(A)** Mutagenesis. **(B)** Additional sensor domain. **(C)** Reconstituting sensor domain, which is divided into chimeric sensor domain and inserting FP into sensor domain.

The sensor domain, which directly interacts with cellular players to induce the conformational change of GEBs, plays a key role in the biosensor’s target sensing ability (Terai et al., 2019). To optimize biosensors, researchers have analyzed the interaction between the sensor domain and target player or ligand domain and devised various strategies around this (Figure 2; Table 1). The first strategy involved mutagenesis (Figure 2A). By mutating one or several residues that play an important role in binding between the sensor domain and its counterpart, the affinity of the sensor domain to target players was regulated. Researchers mainly mutate several residues, but in some cases, the mutation of only one residue had a significant effect on the function of the GEB (Klarenbeek et al., 2015). Moreover, the addition of the same or different sensor domains improves the ability of the biosensor to measure the activities of multiple players simultaneously (Figure 2B). The last method we introduce creates a new sensor domain by combining parts originating from different proteins or inserting another component of the biosensor in the middle of the sensor domain (Figure 2C). Thus, the sensor domain can be reconstructed.

**TABLE 1:** Summary of sensor domain optimization strategies.

Sensor Domain					
Method	Aim	Sensor Name	Target	Original Form	Optimized Form
Mutagenesis	Increasing affinity to target player	Epac-S H187	cAMP	Klarenbeek et al. (2011)	Klarenbeek et al. (2015)
			ZAP70 kinase saFRET biosensor	ZAP70	Li et al. (2016)	Liu et al. (2021)
	Decreasing affinity to target player	Cameleon 3	Ca2+	-	Miyawaki et al. (1997)
		FLIPmal-Y Series	Maltose	-	Fehr et al. (2002)
		ZapCY2	Zn^2+^	Qiao et al. (2006)	Qin et al. (2011)
		Decreasing affinity to non-target player	TN-XL	Ca^2+^	Heim and Griesbeck, (2004)	Mank et al. (2006)
		Increasing affinity to ligand domain	EKAREV	ERK	Harvey et al. (2008)	Komatsu et al. (2011)
	AKAR3EV		PKA	Allen and Zhang, (2006)	Komatsu et al. (2011)
			Imaging at 25°C	ATeam1.03NL	ATP	Imamura et al. (2009)	Tsuyama et al. (2013)
	Additional sensor domain	Increasing affinity to target player	TN-XXL	Ca^2+^	Mank et al. (2006)	Mank et al. (2008)
		Monitoring two targets simultaneously	ICUPID	PKA, cAMP	Dipilato and Zhang, (2009)	Ni et al. (2011)
			KCAP-1	PKC, PKA	Schleifenbaum et al. (2004)	Brumbaugh et al. (2006)
			Reconstituting sensor domain	Creating chimeric sensor domain	Chimera Cx	V^+^	Lundby et al. (2010)	Mishina et al. (2012)
		Inserting FP into sensor domain		FLII^X^Pglu-Y Series	Glucose	Fehr et al. (2003)	Deuschle et al. (2005)

### 2.1 Mutagenesis

#### 2.1.1 Increasing Affinity to Target Player

Biosensors can be optimized by increasing the affinity of the sensor domain to the target molecule. To produce an optimized FRET GEB detecting cyclic adenosine monophosphate (cAMP) with exchange protein directly activated by cAMP1 (EPAC1), Klarenbeek et al. examined several fluorescent protein pairs and residues in the sensor domain (Klarenbeek et al., 2015). In particular, EPAC-S H107 using EPAC1 with Q270E mutation, which causes the sensor domain to have a high affinity for cAMP, showed a 1.6-fold larger FRET ratio change than that of the forerunner having the same FP pair and wild type (WT) EPAC1.

Biosensors that measure kinase activity mainly use truncated peptide sequences, including phosphorylatable residues as the sensor domain. Recently, a case using a platform that combines FRET-seq, a method coupling FRET signals to next-generation sequencing (NGS), and mammalian cell libraries to increase the affinity of a consensus peptide to target kinase was reported (Liu et al., 2021). The first step of this platform was to create a self-activating FRET (saFRET) biosensor by inserting an active kinase domain into the C-terminus of the template biosensor. This process enabled the biosensor to emit an enhanced FRET ratio change in response to external stimuli and reduce the noise signal caused by endogenous cellular kinases. Next, libraries of substrate peptides using the saFRET biosensor were generated. Using degenerate primers, a mix of oligonucleotide sequences covering all possible nucleotide combinations, researchers created two peptide libraries by randomizing neighboring residues of the phosphorylated tyrosine residue: Library 1 (−1, −2, −3, and Y) and Library 2 (Y, +1, +2, and +3). Additionally, the possibility of false-positive selection was reduced by using control libraries of the kinase-dead version of the saFRET biosensor; the control libraries enabled researchers to select substrate peptides that only respond to the target kinase. The saFRET biosensor libraries were then transduced into mammalian cells using viral libraries, and the FRET ratios of individual cells expressing biosensor variants were analyzed and sorted using fluorescence-activated cell sorting (FACS). As a result, with NGS using RNA of the cell emitting a high-FRET ratio, the authors successfully optimized the biosensors that monitor the activity of Fyn and zeta-chain-associated protein kinase 70 (ZAP70) by identifying refined substrate sequences that responded highly to the active kinase domain and lowly to the dead kinase substrates. In this process, substrate peptides with a high affinity to the Src homology 2 (SH2) domain, a ligand domain of the biosensors, were naturally selected. Because this platform screened for improved biosensors using mammalian cells, not bacterial or yeast cells, there was no need to consider differences in translation and post-translational modification depending on the host, and no additional selection steps were required.

#### 2.1.2 Decreasing Affinity to Target Player

Increasing the affinity to the target is not the only way to upgrade the performance of GEBs; researchers have also optimized the sensor domain by reducing the affinity to target molecules. Cameleon3, a genetically encoded calcium indicator (GECI), contains a calmodulin (CaM) in which one component reacting to Ca^2+^ with high affinity was removed via the E104Q mutation (Miyawaki et al., 1997). As a result, the biosensor showed a consistent and simplified sigmoidal curve according to the Ca^2+^ concentration.

The FLIPmal biosensor family detects cellular maltose using maltose-binding protein (MBP) as the sensor domain (Fehr et al., 2002). The primary FLIPmal involving WT MBP had a dissociation constant (K_d_) of 2 μM for maltose. To expand the range of the biosensor for maltose measurements, tryptophan residues of MBP were mutagenized to alanine to decrease substrate affinity (Fehr et al., 2002). FLIPmal-25μ, which has W230A-mutated MBP, showed a K_d_ of 25 μM, and FLIPmal-225μ using W62A MBP had a K_d_ of 226 μM. With these biosensors, researchers have successfully measured various maltose concentration ranges.

The Zap1-based Zn^2+^ probe family uses two Zap1 zinc fingers as the sensor domain. One WT zinc finger detects Zn^2+^ using two cysteines and two histidines (Qiao et al., 2006). The K_d_ of ZapCY1 using two WT zinc fingers for the ion is 2.5 pM (Qin et al., 2011). To measure Zn^2+^ at a higher concentration range, researchers sequentially mutated the cysteines to histidines, and two types of mutation forms were adapted to the biosensor; Cys2His2 mutation was C581H and C618H, and His4 had C581H, C586H, C618H, and C623H mutations. ZapCY2, a Cys2His2 form biosensor, had K_d_ of 811 pM in cell and the K_d_s of ZapCV2 with Cys2His2 mutation and ZapCV5 with His4 mutation for Zn^2+^ were 2.3 nM and 0.3 μM, respectively (Qin et al., 2011). The conversion of more residues to histidine decreased the affinity of the biosensor to Zn^2+^, allowing researchers to measure Zn^2+^ at a high concentration range.

#### 2.1.3 Decreasing Affinity to Non-Target Player

The sensor domain is able to interact with other molecules with properties similar to those of the target players. Therefore, to increase the specificity of the biosensor target, researchers carried out mutations that lower the affinity of the sensor domain to target player analogs. TN-L15, which uses the EF-hand III and IV of chicken skeletal muscle troponin C (csTnC) to detect Ca^2+^, displayed conformational changes and FRET ratio changes in response to Mg^2+^ (Heim and Griesbeck, 2004). Mank et al. improved the specificity of the sensor domain by mutating the D111 and D147 residues of csTnC capable of binding with magnesium into asparagine, leading to increased FRET ratio change and increased sensitive visualization of intracellular Ca^2+^ dynamics (Mank et al., 2006).

#### 2.1.4 Increasing Affinity to Ligand Domain and Altering Reaction Conditions

Depending on which ligand domain is included in the biosensor that measures kinase activity, amino acids around the phosphorylatable residue can be substituted to increase the affinity between the sensor and ligand domains. EKAREV, an extracellular signal-regulated kinase (ERK) activity biosensor, uses the WW domain as a ligand domain to detect phosphothreonine (pThr) (Komatsu et al., 2011). To increase the affinity between the WW domain and substrate peptide, the researcher mutated the pThr +1 position into proline. AKAR3EV, a protein kinase A (PKA) activity biosensor, involves the forkhead-associated 1 (FHA1) domain as a ligand domain for detecting pThr. Consequently, the pThr +3 position was changed to aspartic acid to enhance its affinity to FHA1 (Komatsu et al., 2011).

There is a case of sensor modification using mutagenesis to increase the affinity between the sensor domain and target molecule in a unique experimental environment. AT1.03, which measures intracellular adenosine triphosphate (ATP) status, uses ε subunit of *Bacillus subtilis* FoF1-ATP synthase as sensor domain (Imamura et al., 2009). However, AT1.03 expressed in *Drosophila* S2 cells had poor sensitivity to ATP because of the unique imaging environment with a temperature of 25°C (Tsuyama et al., 2013). In 2013, Tsuyama et al. developed AT1.03NL biosensor by conducting a mutation of M60 in N-terminal domain (NTD) to N and K132 in C-terminal domain (CTD) to L and observed that the biosensor successfully monitored intracellular ATP status in *Drosophila melanogaster* and *Caenorhabditis elegans* at a temperature range is of 20–25°C (Tsuyama et al., 2013).

### 2.2 Additional Sensor Domain

It was reported that a biosensor had two identical sensor domains to increase the affinity to the target molecule and FRET ratio change. In the case of TN-XL, which uses EF-hand III and IV of csTnC as the sensor domain to detect Ca^2+^, the efficiency of the biosensor was improved by adding one more sensor domain, and the advanced biosensor was called TN-XXL (Mank et al., 2008).

There are several cases in which the activity of two targets can be measured simultaneously by including two different sensor domains in one biosensor sequence. ICUPID, a biosensor detecting PKA, and cAMP dynamics, contains three FPs: CFP, red fluorescent protein (RFP), and YFP. (Ni et al., 2011). Between the CFP-RFP pair, there is a PKA substrate peptide and FHA1 domain pair, which can sense PKA activity, and the EPAC1 detecting cAMP was concatenated between the RFP-YFP pair. Therefore, by analyzing the RFP/CFP and YFP/RFP values emitted simultaneously by one biosensor in response to a specific stimulus, the status of intracellular PKA activity and cAMP can be visualized simultaneously. KCAP-1, which was generated by inserting a seven-amino acid PKA consensus sequence (Kemptide) into KCP-1, which measures the activity of protein kinase C (PKC), was designed to monitor the activity of PKC and PKA at the same time (Brumbaugh et al., 2006). In the intermediate FRET ratio state, the ratio increased when PKC was activated, and the FRET efficiency decreased if PKA activity was upregulated. The introduction of additional negative charges by PKA phosphorylation at Kemptide resulted in a disturbance of ionic interaction, inducing reduced FRET efficiency.

### 2.3 Reconstituting the Sensor Domain

In addition to changing specific residues of the sensor domain, there are cases in which a new chimeric sensor domain is created by combining several parts derived from different proteins to enhance the performance of the biosensor. VSFP2.3 is a genetically encodable voltage-sensing fluorescent probe that uses monomeric voltage-sensitive phosphatase (Ci-VSP) originating from *Ciona intestinalis* as the sensor domain (Lundby et al., 2010). In 2012, Mishina et al. transplanted homologous amino acid motifs of mKv3.1, a tetrameric voltage-activated potassium channel, into VSFP2.3 to create a novel chimeric biosensor, the chimeric Cx family (Mishina et al., 2012). Researchers developed Chimera C5 by replacing the Ci-VSP 227–236 amino acid with a counterpart of mKv3.1. Strikingly, Chimera C5 responded faster to activation and deactivation and displayed a higher total response than VSFP2.3.

The performance of the GEB can be improved by inserting an FP in the middle of the sensor domain sequence. The FLIPglu biosensor family, which monitors intracellular glucose, uses the mature glucose/galactose-binding protein MglB from *Escherichia coli* as its sensor domain (Fehr et al., 2003). To optimize the biosensor, Deuschle et al. searched for a site to insert the FP in the MglB sequence (Deuschle et al., 2005). The desired insertion site was required to satisfy the following conditions: it is solvent-exposed, located between regions of well-formed secondary structure, and capable of sterically accommodating the FP. Among the candidates, FLII^12^Pglu-600μ, in which ECFP was inserted after the 12th amino acid of MglB, showed the most improved FRET ratio change.

## 3 Ligand Domain

**FIGURE 3:**
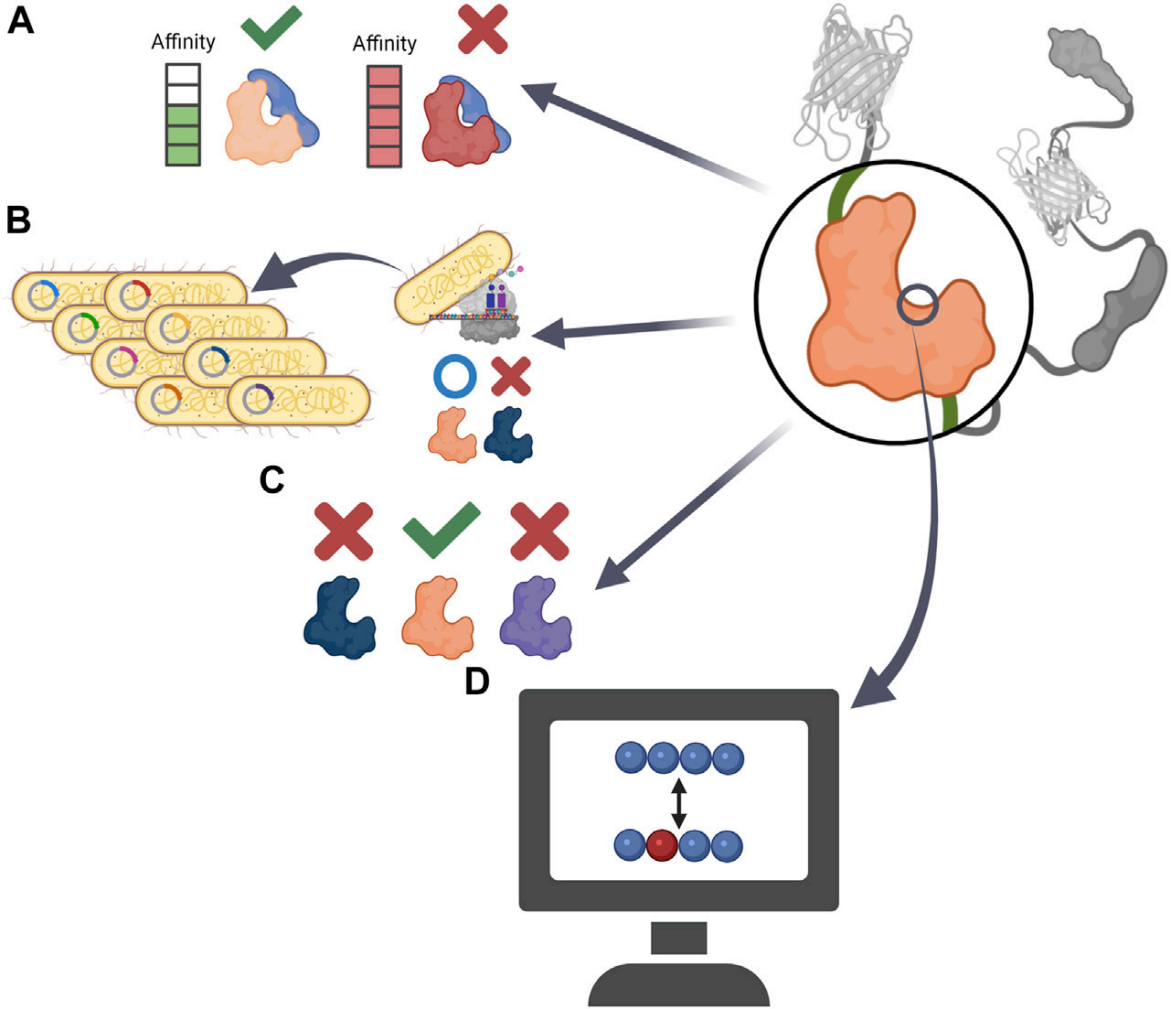
Schematic precedents of ligand domain optimization **(A)** Constructing a GEB that can reversibly respond to external stimuli by using ligand domain with adequate affinity to sensor domain. **(B)** By replacing the ligand domain that *Escherichia coli* could not express, the GEB could be applied to a high-throughput bacterial colony screen **(C)** Developing target specific GEB by comparing the performance of ligand domains derived from various proteins. **(D)** Creating new ligand domain having low affinity to endogenous player via computational analysis.

The primary form of GEB consists of only the FRET pair and sensor domain, and the emitted FRET ratio changes only due to conformational changes in the sensor domain (Terai et al., 2019). However, for a more dynamic conformational change of the biosensor, researchers have begun to incorporate a ligand domain, which detects the change in the sensor domain, into the biosensor structure. Therefore, the introduction of the ligand domain itself is an optimization of the GEBs (Terai et al., 2019). There are various types of ligand domains depending on the target players, but this review focuses on cases in which the ligand domain has been upgraded (Figure 3; Table 2).

**TABLE 2:** Summary of ligand domain optimization strategies.

Ligand Domain					
Method	Aim	Sensor Name	Target	Original Form	Optimized Form
Changing to ligand domain having appropriate affinity	Making biosensor reversible for detecting bi-directional PKA related signal	AKAR2	PKA	Zhang et al. (2001)	Zhang et al. (2005)
Changing to the ligand domain that *Escherichia coli* can translate	To apply the GEB to high-throughput bacterial colony screen	CyclinB1-Cdk1 activity sensor V2	Cdk1	Gavet and Pines, (2010)	Belal et al. (2014)
Comparing ligand domain homologies	Finding PDGFR activity-specific ligand domain	PDGFR biosensor	PDGFR	-	Seong et al. (2017)
Mutagenesis based on computational analysis	Decreasing affinity with endogenous players	Design X	Ca^2+^	Griesbeck et al. (2001)	Palmer et al. (2004), Palmer et al. (2006)

### 3.1 Detecting Phosphorylated Residue

AKAR, which measures the activity of PKA, uses 14-3-3 protein as a ligand domain to capture pThr (Zhang et al., 2001). However, the binding affinity of 14-3-3 to pThr was too strong, preventing dephosphorylation of phosphorylated residues, which made it difficult for AKAR to monitor the effect of intracellular phosphatase on PKA substrate peptide. Four years later, AKAR2, the successor to AKAR, used FHA1 as its ligand domain (Zhang et al., 2005). FHA1, a modular phosphothreonine binding domain with relatively low binding affinity, did not interfere with the dephosphorylation of pThr and made AKAR2 a reversible reporter detecting the bi-directional state of PKA substrate (Figure 3A).

There is a case in which the ligand domain was replaced with another protein for a transfected host to express the biosensor well. For GEB optimization using high-throughput bacterial colony screening, Belal et al. attempted to express the cyclin B1-Cdk1 activity sensor in *Escherichia coli* (Belal et al., 2014). However, the biosensor was not translated well in *E. coli* because of the polo box domain of Plk1, a ligand domain of the biosensor. Therefore, the ligand domain was replaced with FHA2, and the improved biosensor was well expressed in bacteria and could be applied to the screening platform (Figure 3B).

The FHA family is not the only family used to detect phosphothreonine. In the case of the EKAR family measuring the activity of ERK, the WW domain has been used as a ligand domain since 2008, which continues to be utilized, and EKAREN4/5, which has high specificity for ERK but low for CDK1, was developed in 2021 (Harvey et al., 2008; Ponsioen et al., 2021). Therefore, an appropriate ligand domain should be examined according to the substrate peptide of the target player.

The SH2 domain, which detects phosphotyrosine (pTyr), can be derived from various proteins (Nair et al., 1995; Songyang and Cantley, 1995). Each SH2 domain has a different preferred environment and affinity for pTyr, which may affect the performance of the GEB. To develop a FRET-based platelet-derived growth factor receptor (PDGFR) biosensor measuring PDGFR phosphorylation, Seong et al. examined the SH2 domains derived from Src, Nck2, and Shp2 (Seong et al., 2017). The biosensor using the SH2 domain of Src showed conformational change and FRET ratio change not only by activated PDGFRs but also by other kinases, meaning that the SH2 domain originating from Src did not have specificity for PDGFR activity. However, the biosensor containing the SH2 domain of Nck2 and Shp2 displayed a PDGFR activity-specific FRET ratio change, and the SH2 domain derived from Nck2 efficiently improved the performance of the PDGFR biosensor located in the plasma membrane. Therefore, when developing or optimizing a biosensor to monitor the activity of protein tyrosine kinases, it is important to examine various types of SH2 ligands (Figure 3C).

### 3.2 Reducing Affinity With an Endogenous Player: For Precise Ca^2+^ Monitoring

Biosensors can be optimized by preventing the sensor and ligand domains from interacting with their endogenous cellular partners (Figure 3D). Because the WT ligand domain, a skeletal muscle myosin light chain kinase (skMLCK), of primary cameleons, a GECI using CaM as the sensor domain, could interact with endogenous CaM, cameleons failed to accurately respond to the Ca^2+^ concentration in the CaM-rich regions such as the plasma membrane of neurons (Griesbeck et al., 2001; Heim and Griesbeck, 2004). To create an improved Cameleon that does not bind to endogenous CaM, Palmer et al. structurally analyzed and mutated the sensor and ligand domains in the GEB and called the optimized CaM-skMLCK pairs ‘Design X’. In 2004, the salt-bridge interaction between WT CaM and WT skMLCK peptides was investigated to develop Design 1 (D1) (Palmer et al., 2004). To break the salt-bridge between skMLCK of cameleon and WT CaM, the basic target residues of skMLCK peptide and acidic residues of CaM were reversed, thus preventing the biosensor components from binding with their WT counterparts. As a result, the researchers successfully developed D1, which has a relatively low affinity for Ca^2+^ (K_d_ = 60 μM) and was not significantly affected by large concentrations of excess CaM. In 2006, Designs 2, 3, and 4 were released (Palmer et al., 2006). To destabilize the binding between WT CaM and skMLCK peptide in the Cameleon in other ways, the researchers analyzed steric bumps in WT skMLCK and complementary holes in WT CaM. Based on this study, a small but important residue that plays a key role in WT CaM-WT skMLCK interaction was replaced with bulkier or charged residues, resulting in the development of mutated versions of skMLCK (mskMLCK), which sterically clashed with WT CaM. Thereafter, by mutating the CaM residues that interacted with WT skMLCK, the researchers created mutated versions of CaM (mCaM) that bound well to mskMLCK peptide but not to WT skMLCK. In this way, three combinations of mCaM-mskMLCK, which bound weakly to WT CaM but interacted much more strongly than the WT CaM-WT skMLCK pair, were created and named D2, D3, and D4. The new Designs could sensitively measure various ranges of Ca^2+^ concentrations that had not been monitored before and successfully visualize calcium in cellular microenvironments with unique Ca^2+^ concentrations, such as in the lipid rafts and mitochondria.

## 4 Linker

**FIGURE 4:**
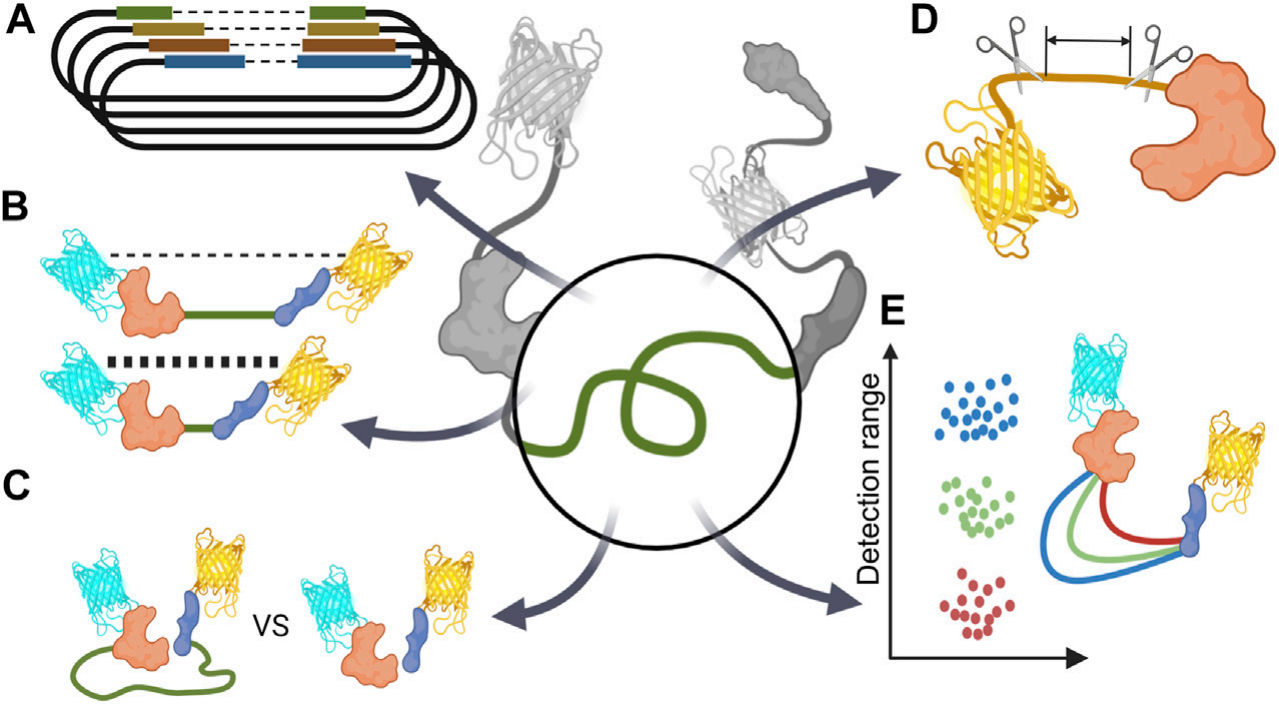
Schematic diagram of linker optimization strategy **(A)** Optimize linker length via vector library. **(B)** Minimize basal FRET signal by extend linker **(C)** Comparing unimolecular and bimolecular biosensors. **(D)** Reducing unintentional linker-like peptides such as protein terminal parts or short peptide originated from restriction enzyme DNA sequence. **(E)** Changing sensor detection range via linker optimization.

GEBs are chimeric proteins in which peptide sequences derived from various proteins are collected in one amino acid chain. Therefore, a connection inevitably exists between each component, which is called a linker. Because these linkers have a significant influence on the physical structure of GEBs, there have been various attempts at their optimization thereof (Figure 4; Table 3).

**TABLE 3:** Summary of linker optimization strategies.

Linker					
Method	Aim	Sensor Name	Target	Original Form	Optimized Form
Testing 49 combinations of proline linkers via vector library	Optimize linker length	Twitch	Ca^2+^	Mank et al. (2008)	Thestrup et al. (2014)
Add rigid linker	Linker optimization for reducing basal FRET signal	CY-RL7	redox status	Kolossov et al. (2008)	Kolossov et al. (2011)
Insert EV linker		AKAR3EV	PKA	Allen and Zhang, (2006)	Komatsu et al. (2011)
		AMPKAREV	AMPK	Tsou et al. (2011)	Konagaya et al. (2017)
Add GSGGPPGSGGSG Linker, 58a.a linker and 10a.a Linker		NIR Rac1	Rac1	Miskolci et al. (2016); Moshfegh et al. (2014)	Shcherbakova et al. (2018)
Extend EV linker from 116a.a to 244a.a		Booster-PKA	PKA	Komatsu et al. (2011)	Watabe et al. (2020)
Insert flexible linker	Unimolecular biosensor VS. bimolecular biosensor	FLAME/PTB-EYFP, EGFR-ECFP	EGFR	-	Offterdinger et al. (2004)
Add (GGSGGS) repeat sequence		CA-L2-WY	Zn^2+^	Van Dongen et al. (2006)	Van Dongen et al. (2007)
Add or remove EV linker		KARs/bimKARs	ERK	-	Depry et al. (2015)
Delete a.a sequence of FPs N/C terminus and sensor domain C terminus	Optimization for reducing unintentional linker-like peptides	FLIPglu-600u Δ13	Glucose	Fehr et al. (2003)	Deuschle et al. (2005)
Delete a.a sequence of restriction enzyme site		FLII^12^Pglu-δ6		Deuschle et al. (2005)	Takanaga et al. (2008)
Add glycine or serine	Changing sensor detection range	YC-Nano	Ca^2+^	Nagai et al. (2004)	Horikawa et al. (2010)
Adjust repeats from (GPGGA)_8_ to (GPGGA)_5_		TSMod F25	Mechanical strain	Grashoff et al. (2010)	Brenner et al. (2016)
Replace (GPGGA)_8_ linker with HP35 linker		HP35-TS		Grashoff et al. (2010)	Austen et al. (2015)
SDM at N68A, K70M of HP35-TS		HP35st-TS		-	Austen et al. (2015)

Initially, the linker optimization process improved the FRET ratio by changing the direction of the fluorescent protein through various amino acid changes between FPs and the Ca^2+^-sensing domains, while Miyawaki et al. developed the Cameleon1 biosensor (Miyawaki et al., 1997). Because of these studies, various attempts have been made on linker optimization.

There are various types of linkers depending on the purpose of the linker, but they can be broadly divided into flexible and rigid linkers (Gräwe and Stein, 2021).

The flexible linker has the feature of free movement between proteins linked by a linker that does not have a specific structure. A typical example is glycine-rich linkers. Because glycine has a small side chain, it was considered optimal for folding, structure, and function of the GEB linker (Chen et al., 2013). If the linker is flexible, the relative orientation between the donor and acceptor proteins changes. Therefore, the orientation factor, *k* ^2^, which is related to the FRET ratio, was set to ⅔. Therefore, the FRET efficiency can be calculated using the distance between the donor and acceptor without considering the variation in the orientation factor between fluorescent proteins (Terai et al., 2019).

The rigid linker is a linker with a specific structure and usually has an α-helical structure, which can be adjusted to prevent free interactions between the two connected proteins (Swanson and Sivaramakrishnan, 2014). A typical example is the (EAAAK)_N_-motif. The α-helical structure has been applied in FRET protein sensors to monitor glycine (Zhang et al., 2018), redox potential (Kolossov et al., 2011), and ionic strength (Liu et al., 2017). If the linker is rigid, because of the fixed distance, during folding or shape changes in the linker due to sensor activation or environmental conditions such as ionic interactions (Liu et al., 2017) or the oxidation status of disulfide bridges (Kolossov et al., 2011), a high FRET ratio change can be obtained.

The linker affects the three-dimensional physical structure of the biosensor, and it is possible to manage the binding between the sensor and ligand domains. Here we introduce cases that affect the efficiency of the FRET sensor by optimizing the linker.

### 4.1 Optimize Linker Length

There was a case in which various combinations of linkers were optimized through a vector library (Figure 4A). Twitch, a calcium indicator, has a sensor domain, and TnC is concatenated between the donor and acceptor. Thestrup et al. used a vector library to determine the optimal number of proline residues that connect the sensor domain N/C terminal and each fluorescent protein. They revealed the linker combination with the highest FRET efficiency by comparing a total of 49 linker combinations, from 1 to 7 prolines, and the front and back of the sensor domain (Thestrup et al., 2014).

### 4.2 Linker Optimization for Reducing Basal FRET Signal

In most cases of FRET-based biosensors, they are mainly used as ratio metrics through fractional calculations of donor and FRET signals. Therefore, if the difference between the basal FRET ratio before the reaction and the FRET ratio after the reaction is significant, it can be said that the FRET biosensor has high sensitivity. To increase this difference, the linker can be optimized to increase the FRET signal after reaction; on the contrary, the linker can be optimized to have a low basal FRET signal when the reaction does not occur to increase the difference between the basal FRET signal and the activated FRET signal (Figure 4B).

The use of a rigid linker to reduce the basal FRET signal is the CY-RL7 redox sensor (Kolossov et al., 2011). In the CY-RL5 biosensor structure (Kolossov et al., 2008), the RL5 linker, which is sensitive to redox status, is located between the CFP-YFP pair; when the linker is reduced (OFF state), the α-helix structure of the linker is changed, and the distance between the CFP-YFPs is increased, which leads to a decrease in the FRET signal. Conversely, when the linker is oxidized (ON state), the distance between the two fluorescent proteins decreases, resulting in an increase in the FRET signal. To improve the FRET ratio change, Kolossov et al. added the EAAAK rigid linker at the RL5 linker to reduce the distance between the donor and acceptor. They observed a six-fold change in FRET efficiency (Kolossov et al., 2008) and named this newly optimized sensor CY-RL7.

The cases of using a flexible linker to reduce basal level FRET signal are the AKAR3EV (Komatsu et al., 2011) and AMPKAREV (Konagaya et al., 2017) biosensors that use EV linker. The EV linker is a flexible linker consisting of repetitive SAGG peptides developed by Matsuda Lab. AKAR3 (Allen and Zhang, 2006) and AMPKAR (Tsou et al., 2011) biosensors have donor and acceptor at both ends, and ligand domains and sensor domains are attached between them. In Matsuda Lab, EV linkers were added between domains to increase the distance between the donor and acceptor. As a result, the basal FRET signal was reduced, thereby improving the FRET efficiency of the biosensor. They named the newly optimized sensors AKAR3EV and AMPKAREV, respectively.

Because the linker optimization process is closely related to the three-dimensional structure of the biosensor, if each part of the sensor is shuffled or replaced with another protein in the existing sensor, the basal FRET ratio will change owing to the change in the physical structure. Therefore, it is necessary to optimize the linker accordingly.

In the case of the NIR-Rac1 (Shcherbakova et al., 2018) biosensor using the miRFP670-miRFP720 FRET pair, the Forster radius (R_0_) value of the fluorescent protein pair was 8.3 nm, 1.5–1.7 times longer than that of the normal CFP-YFP pair. Therefore, a relatively longer linker was used to separate fluorescent proteins.

In the case of the Booster-PKA (Watabe et al., 2020) biosensor, Watabe et al. arranged the PKA substrate at the C-terminus while designing the biosensor, so that the distance between the donor and the acceptor is closest when the sensor responds. However, this structure allowed the distance between the donor and acceptor to be close in the basal state. Therefore, to increase the sensitivity of the sensor, the length of the previously constructed EV linker was extended from 116 a. a to 244 a. a and the distance between fluorescent proteins was increased to lower the basal FRET signal.

### 4.3 Unimolecular Biosensor VS. Bimolecular Biosensor

In the case of a linker-connected type, unimolecular biosensor (UnimB), because the ligand and sensor domains move together at a relatively constant distance, the sensor response is more constant than that in the separated type, bimolecular biosensor (BimB), in which the distance between domains is not constant and the possibility of interaction with endogenous proteins induces the noise in the FRET signal. In addition, because the unimolecular Biosensor is expressed from single vector, accurate measurement of FRET is possible because all compartments show the same expression level and are located in the same region (Kim et al., 2021) (Figure 4C).

There are cases where the optimization was performed as UnimB using a linker because BimB did not react. An increase in the emission intensity of the acceptor by EGF treatment was observed when the PTB-EYFP/EGFR-ECFP bimolecular (Offterdinger et al., 2004) biosensors were used to measure EGFR autophosphorylation; however, there was no change in the FRET ratio observed. However, FLAME (Offterdinger et al., 2004), in which the two constructs were combined through a linker, successfully changed the FRET ratio via EGF stimulation.

To optimize the BimB Zn^2+^ indicator, CFP-Atox1 and WD4-YFP, Van Dongen et al. compared the FRET ratio in each case by inserting two to nine GGSGGS repeat sequences between Atox1 and WD4, the metal-binding domains. Each biosensor had a unique K_d_ value and FRET ratio change range. In particular, CA-L2-WY containing (GGSGGS)_2_ showed the most remarkable FRET ratio change upon Zn^2+^ stimulation (Van Dongen et al., 2007).

However, UnimBs were not advantageous in all cases. Depending on the characteristics of the ligand domain and the sensor domain or the location of the sensor, BimB showed a better FRET ratio in some cases than in others.

The efficiencies of UnimB and BimB types of kinase activity reporters that measure various kinase activities were compared, and the change in efficiency was found to be different for each biosensor. There were even cases where it did not change (Depry et al., 2015). These differences depended on the target location and the location of the sensor. In particular, the biosensor placed on the plasma membrane showed improved FRET ratio values in BimB (Depry et al., 2015).

### 4.4 Linker Optimization for Reducing Unintentional Linker-like Peptides

When constructing a sensor for the first time, WT proteins are usually used. WT proteins have the advantage of reliability in the operation of the sensor by showing a physiologically similar reaction to that of the proteins present in the living body. However, most WT proteins have several peptide sequences, each possessing a terminal region. These peptide sequences unintentionally affect the operation of the biosensor, or even though these sequences have no function, they act as linkers. Therefore, these peptide sequences could also be optimized (Figure 4D).

It may be necessary to consider the sequence between the sensor components for sensor optimization. To optimize the FLIPglu-600u (Fehr et al., 2003) biosensor, which is one of the FLIPglu biosensor family that measures the intracellular glucose concentration, the amino acid sequence of the N/C-terminus part of the fluorescent protein (Li et al., 1997), and the C-terminus part of the sensor domain was removed. The authors named the cloned sensor FLIPglu-600u Δ13 (Deuschle et al., 2005). This sensor showed a FRET ratio change of approximately threefold. In addition, the authors cloned the FLII^12^Pglu (Deuschle et al., 2005) biosensor in which a fluorescent protein was inserted between the sensor domains to optimize FLIPglu-600u Δ13. In the optimization process of this sensor, a restriction enzyme sequence occurred during the cloning process, and the FLII^12^Pglu-δ6 (Takanaga et al., 2008), which removed this sequence, showed the highest efficiency. However, the FRET ratio was more influenced by the composition of the removed amino acid than by the length of the linker removed (Takanaga et al., 2008).

### 4.5 Linker Optimization for Changing Sensor Detection Range

Linker optimization sometimes improved the detection range of the biosensor rather than improving the FRET ratio (Figure 4E).

There is a case where the detection range was changed by changing the sequence of several amino acids in the linker. The genetically encoded calcium indicator (GECI), YC2.60, and YC3.60 (Nagai et al., 2004), had a structure in which the CaM and M13 domains were connected through two glycines. Horikawa et al. added glycine or serine to the linker to diversify the saturation concentration of calcium by changing the sensitivity of the sensor to calcium. This helped develop the YC-Nano family, a collection of sensors that can measure various calcium concentration ranges (Horikawa et al., 2010).

Tension-sensing FRET biosensors are biosensors developed to measure various tensions within cells. To measure the physical force, these types of sensors use elastic linkers. Therefore, the linker itself acts as a force-sensing sensor domain. By modifying these linkers, researchers have diversified the range of force measurements.

In the case of TSMod F40 (Grashoff et al., 2010), mechanical strain in the range of 1‒6 pN in the cell could be measured using the flagelliform linker sequence (GPGGA)_8_, which is an elastic linker derived from spider silk protein. To adjust the force measurement range, Brenner et al. developed TSMod F25 and TSMod F50 (Brenner et al., 2016), in which the repeat number of the GPGGA sequence was adjusted to 5 and 10, respectively, and the measurable range was compared with that of TSMod F40. As a result, the TSMod F25 containing a (GPGGA)_5_ linker could have an improved force measurement range of 2‒11 pN compared to that of the TSMod F40.

To adjust the force measurement range, the linker was substituted with other protein types. Austen et al. produced HP35-TS (Austen et al., 2015) by replacing the linker of the TSMod F40 biosensor with villin headpiece peptide (HP35), an ultrafast folding peptide. The HP35-TS was able to measure the force range of 6‒8 pN, which is stronger than the TSMod F40 with a measuring range of 1‒6 pN. In addition, HP35st-TS (Austen et al., 2015) containing a more stable folding peptide was produced through N68A and K70M mutations in HP35-TS, which can measure a stronger force range of 9–11pN.

## 5 Localization Signal

**FIGURE 5:**
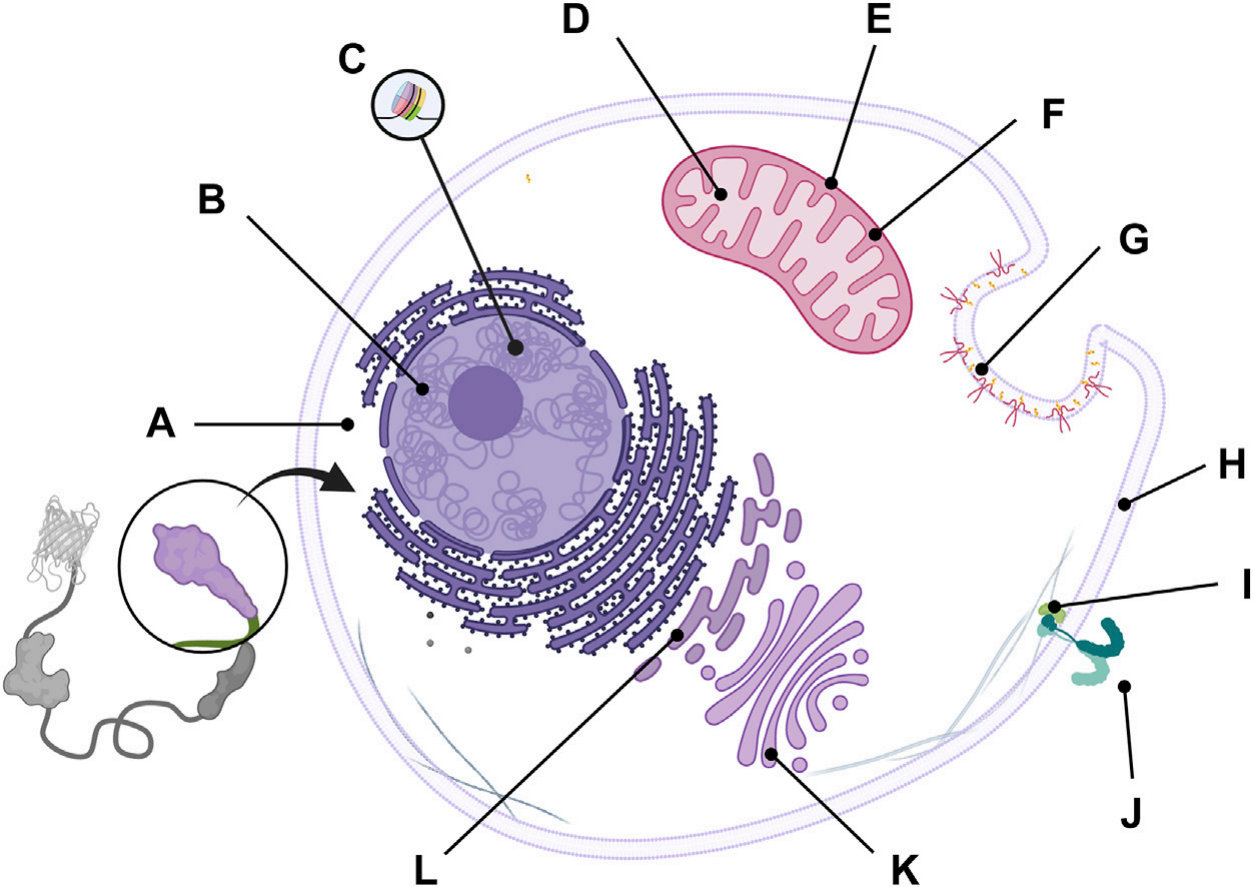
Representative localization signal to guide GEBs at specific cellular microdomain. The biosensors can be placed at **(A)** cytosol by attaching nuclear export signal (NES), **(B)** nucleus by including nuclear localization signal (NLS), **(C)** a nucleosome subunit by using Histone H3, **(D)** mitochondrial matrix by attaching mitochondrial targeting sequence (MTS), **(E)** mitochondrial outer membrane by containing C-terminal sequence of Bcl-xl, **(F)** mitochondrial intermembrane region by including leader sequence from the second mitochondria derived activator of caspases (SMAC), **(G)** lipid raft by using Lyn acylation substrate sequences, **(H)** nonraft by attaching K-Ras prenylation sequences, **(I)** IRS-1 working site by using PH and PTB domain of IRS-1, **(J)** Extracellular region by including Igκ-chain leader sequence and PDGFR transmembrane domain, **(K)** Golgi apparatus by attaching eNOS targeting domain, and **(L)** endoplasmic reticulum (ER) by containing calreticulin signal sequence (CRsig) and ER retention sequence (KDEL).

Most target players in cells exist naturally in specific subcellular locations, depending on their role. For example, during the cell adhesion process of human mesenchymal stem cells, Ca^2+^ and focal adhesion kinase (FAK) activities depend on the plasma membrane microdomain (Kim et al., 2019). Additionally, the same player exists at different concentrations depending on where they are. The concentration of local endogenous CaM near the mouth of the channels was higher than that of cytoplasmic CaM (Mori et al., 2004). In addition, calcium is present at 100 nM‒2 µM concentrations in the cytosol, 100–800 µM in the endoplasmic reticulum, and 100 nM‒500 µM in the mitochondria (Arnaudeau et al., 2001; Samtleben et al., 2013). Therefore, to accurately monitor the physiology of a target molecule in a specific region, researchers need to anchor their GEB to the site where the biosensor can adequately interact with the cellular player by using a localization signal (LS). With this strategy, researchers were able to successfully visualize the activity of a target player, which was difficult to detect using a biosensor located at an inappropriate site (Kim et al., 2019). We will introduce examples of successful monitoring of site-specific target player activity or increased FRET ratio change using various LSs. Representative LSs for locating biosensors in subcellular regions are summarized in Figure 5 and Table 4.

**TABLE 4:** Summary of representative localization signals.

Localization Signal			
Localization Signal	Location	Sensor Name	References
Nuclear export signal (NES)	Cytosol	EKAREV	Komatsu et al. (2011)
Nuclear localization signal (NLS)	Nuclear	JNKAR1EV-NLS	Kim et al. (2020)
Histone H3	Histone H3	H3K9me3 Biosensor	Peng et al. (2018)
Mitochondrial targeting sequence (MTS)	Mitochondrial matrix	mito-ZifCY1.173	Park et al. (2012)
C-terminal sequence of Bcl-xl	Mitochondrial outer membrane	Daglas-mit1	Sato et al. (2006)
Leader sequence from the second mitochondria derived activator of caspases (SMAC)	Mitochondrial intermembrane region	SMAC-mCherry-GZnP2	Fudge et al. (2018)
Lyn acylation substrate sequences	Lipid raft	Lyn-Src biosensor	Seong et al. (2009)
K-Ras prenylation sequences	Nonraft	Kras-Src biosensor	Seong et al. (2009)
PH and PTB domain of IRS-1	IRS-1	Phocus-2ppnes	Sato et al. (2002)
Igκ-chain leader sequence and PDGFR transmembrane domain	Extracellular region	MT1-MMP biosensor	Ouyang et al. (2008)
eNOS targeting domain	Golgi apparatus	eNOS-Aktus	Sasaki et al. (2003)
Calreticulin signal sequence (CRsig) and ER retention sequence (KDEL)	Endoplasmic reticulum (ER)	ER Ca^2+^ sensor	Kim et al. (2017)

Some LSs can place biosensors on specific histone proteins (Figure 5C). By positioning the H3K9me3 biosensor precisely at the H3 position of histone with histone H3 sequence in the C-terminus of the GEB, the biosensor successfully monitored the interaction between H3 and players regarding K9H3 methylation and showed an improved FRET ratio change compared to the K9 reporter, a previous version without Histone H3 LS (Lin et al., 2004; Peng et al., 2018).

Several LSs are located in biosensors at specific mitochondrial regions. Park et al. developed mito-ZifCY1.173, which can monitor the Zn^2+^ concentration in the mitochondrial matrix by inserting a mitochondrial targeting sequence (MTS) into the N-terminal of ZifCY1 (Park et al., 2012) (Figure 5D). In addition, Sato et al. successfully analyzed diacylglycerol (DAG) dynamics in the outer mitochondrial membrane by attaching the C-terminal sequence of Bcl-xl to Daglas, a GBE sensing DAG (Sato et al., 2006) (Figure 5E).

A variety of LSs can position GEBs in specific plasma membrane microdomains. To visualize Src kinase activity according to the plasma membrane compartment, a FRET-based Src biosensor was located in the lipid raft or non-raft region by using Lyn acylation substrate sequences and K-Ras prenylation sequences, respectively (Seong et al., 2009) (Figures 5G,H). Interestingly, researchers observed that Src in the lipid raft region responded much more slowly and weakly to growth factors or pervanadate than non-raft domains. With visualization of drugs breaking the cytoskeletal structure, it was revealed that Src in the non-raft region is at rest and is activated immediately, but another Src population in the lipid raft domain responds relatively slowly to external stimuli because it is in an endosome-like structure near the nucleus. In addition, GEBs can radiate increased FRET ratio changes by using a player’s endogenous LS. Phocus, a biosensor that can measure insulin receptor activity in response to insulin treatment, could be situated near the insulin receptor by including a pleckstrin-homology (PH) domain and a phosphotyrosine-binding (PTB) domain, both of which are derived from insulin receptor substrate-1 (IRS-1) in the N-terminal and nuclear export signal (NES) in the C-terminal (Sato et al., 2002) (Figure 5I). Consequently, the Phocus behaved similar to an IRS-1 and was located next to the insulin receptor, inducing more phosphorylation opportunities in the biosensor. As a result, the GEB showed a larger FRET ratio change than the same biosensor in the cytoplasm.

To visualize the activity of the target molecule in the extracellular region, researchers have inserted the Igκ-chain leader sequence in the N-terminus and PDGFR transmembrane domain in the C-terminus of biosensors (Figure 5J). Ouyang et al. successfully analyzed the extracellular activity of membrane type 1 matrix metalloproteinase (MT1-MMP), which remodels the extracellular matrix (Ouyang et al., 2008), and Hires et al. detected glutamate on the surface of cultured dissociated hippocampal neurons (Hires et al., 2008).

Unintentionally embedded LS in GEB components can negatively affect the performance of biosensors. The ICUE biosensor contained the full sequence of EPAC1 to detect cAMP, inducing the biosensor to be situated in the mitochondria or membrane because of the N-terminal region and DEP domain of EPAC1 (DiPilato et al., 2004). Therefore, ICUE is perturbed by endogenous cellular functions and cannot detect the target signal well. ICUE2, a successor to ICUE, used truncated EPAC1 as its sensor domain to exclude the endogenous LSs and to improve the shortcomings, and the biosensor could be evenly distributed in the cytoplasm (Violin et al., 2008). Therefore, when designing a biosensor, it is necessary to check whether the unintentionally included LS lies within the GEB amino acid sequence.

There are more types of LS than we have introduced in this review. To measure the activity of a target player in a cellular region that has not been investigated before, the LS of a protein in the area can be applied to the biosensor.

## 6 Discussion

The biosensors improved by these optimization tactics can not only visualize the dynamics of target players more precisely and sensitively but also be applied to several prospective fields. For example, an optimized FRET-based biosensor can be applied to high-throughput drug screening (HTDS) (Liu et al., 2021). Drug screening using biosensors makes it easy for researchers to obtain high-dimensional experimental results and real-time measurements of the drug’s effect on target molecules in living cells. The previously introduced ZAP70 kinase saFRET biosensor showed increased FRET ratio change in response to drug stimulation by including an active kinase domain and met the conditions to be applied to HTDS, and the dynamic range of the FRET-based GEB must exceed at least 20% (Inglese et al., 2007). Using the optimized FRET-based biosensor, the 96-member kinase inhibitor library was screened, and three potential ZAP70 inhibitors were identified.

By adding the function of the “activator” to the FRET-based GEB, it is possible to visualize the activity of the target player and manipulate the physiology of cells simultaneously. Sun et al. created the Shp2-integrated sensing and activating protein (Shp2-iSNAP), which not only detects the phosphorylation of SIRPα, a receptor of CD47 transmitting “don’t eat me” signal but also inhibits the SIRPα downstream signal with protein tyrosine phosphatase included in biosensor sequence (Sun et al., 2017). When bone marrow-derived macrophages (BMDMs) expressing the biosensor were stimulated by tumor cells that highly express CD47, researchers observed the phosphorylation of SIRPα. However, the macrophage cells engineered by protein tyrosine phosphatase in the biosensor engulfed the cancer cells despite the interaction between BMDMs and CD47. In this way, the biosensor combined with an activator can be used for therapeutic purposes by reprogramming the cells.

In this review, we introduced examples where researchers have optimized biosensor components independently of FPs, and these are summarized in Tables 1–4. To upgrade GEBs, researchers adopted various optimization strategies depending on the target players and experimentally confirmed the tactics. As reported in this paper, researchers not only increased the affinity between the sensor and ligand domains, but paradoxically decreased the affinity to enhance the function and activity of GEB. Meanwhile, it should be taken into account that optimization strategies belonging to one category can upgrade other GEB compartments. For example, a method meliorating sensor domain could be utilized to improve the ligand domain. In addition, to optimize GEBs, the physical structure of GEB’s compartments and experimental temperature were even considered. In this paper, we have presented methods to optimize GEB through various examples, but variables still exist, so it may be necessary to try optimization through various approaches depending on the situation of each researcher. Nevertheless, we hope that the various GEB optimization factors presented in this paper will inspire researchers who want to develop new types of optimized GEBs or improve the performance of existing biosensors.

## Glossary

GEB: genetically encoded biosensor
FP: fluorescent protein
WT: wild type
saFRET: self-activating FRET
ZAP70: zeta-chain-associatedproteinkinase70
SH2: Src homology 2
GECI: genetically encoded calcium indicator
MBP: maltose binding protein
csTnC: chicken skeletal muscle troponinC
pThr: Phosphothreonine
FHA1: forkhead-associated
NTD: N-terminal domain
CTD: C-terminal domain
Ci-VSP: monomeric voltage-sensitive phosphatase
pTyr: phosphotyrosine
skMLCK: skeletal muscle myosin light chain kinase
mskMLCK: mutated versions of skMLCK
mCaM: mutated versions of CaM
BimB: bimolecular biosensor
UnimB: unimolecular biosensor
a.a: amino acid
bimKARs: bimolecular kinase activity reporters
KARs: kinase activity reporters
SDM: site-directed mutagenesis
MTS: mitochondrial targeting sequence
LS: localization signal
NES: nuclear export signal
HTDS: high throughput drug screening
Shp2-iSNAP: Shp2-integrated sensing and activating protein
BMDMs: bone marrow-derived macrophages
PTB: phosphotyrosine-binding
PH: pleckstrin-homology
